# ERRATUM: Nursing workload in hematopoietic stem cell transplantation: a cohort study

**DOI:** 10.1590/1980-220X-REEUSP-2024-ER13en

**Published:** 2024-10-04

In the article “Nursing workload in hematopoietic stem cell transplantation: a cohort study”, with the DOI: https://doi.org/10.1590/S0080-623420150000700014, published by the journal: “Revista da Escola de Enfermagem da USP, volume 49 (spe) of 2015, p. 92–98, on page 98:

**Where it was written:**

## Financial support

Conselho Nacional de Desenvolvimento Científico e Tecnológico (CNPQ). Process number 480388/2012-1.

**Now read:**

## Financial support

Conselho Nacional de Desenvolvimento Científico e Tecnológico (CNPQ). Process number 480388/2012-1. This study was financed in part by the Coordenação de Aperfeiçoamento de Pessoal de Nível Superior – Brasil (CAPES) – Finance Code 001.

