# Supplementary material for: ERRATUM: Nursing workload in hematopoietic stem cell transplantation: a cohort study
Source: Rev Esc Enferm USP. 2024 Oct 4;58:e2024ER13. doi: 10.1590/1980-220X-REEUSP-2024-ER13en (PMC11460655; doi:10.1590/1980-220X-REEUSP-2024-ER13en)
Supplement: Translation

# Translation: ERRATA: Carga de trabalho de enfermagem em transplante de células-tronco hematopoiéticas: estudo de coorte

**DOI:** 10.1590/1980-220X-REEUSP-2024-ER13pt

No artigo “Carga de trabalho de enfermagem em transplante de células-tronco hematopoiéticas: estudo de coorte”, com número de: DOI: https://doi.org/10.1590/S0080-623420150000700014, publicado na Revista da Escola de Enfermagem da USP, volume 49 (spe) de 2015, p. 93–100, na página 100:

**Onde se lia:**

## Apoio financeiro:

Conselho Nacional de Desenvolvimento Científico e Tecnológico (CNPQ). Projeto n. 480388/2012-1. 

**Leia-se:**

## Apoio financeiro

Conselho Nacional de Desenvolvimento Científico e Tecnológico (CNPQ). Projeto n. 480388/2012-1O. O presente trabalho foi realizado com apoio da Coordenação de Aperfeiçoamento de Pessoal de Nível Superior – Brasil (CAPES) – Código de Financiamento 001.
